# Aspirin Use and Liver-Related Outcomes in Metabolic Dysfunction-Associated Steatotic Liver Disease: A Systematic Review and Meta-Analysis

**DOI:** 10.3390/biomedicines14061249

**Published:** 2026-05-30

**Authors:** Fares Jamal, Abdullah Hamad, Amani Elshaer, Pierce L. Claassen, Taylor Viggiano, Michele Barnhill, David M. H. Chascsa, Hugo E. Vargas, Bashar A. Aqel, Blanca C. Lizaola-Mayo

**Affiliations:** 1 Division of Hematology & Oncology, Mayo Clinic, Phoenix, AZ 85054, USA; jamal.fares@mayo.edu; 2 Division of Pulmonary Medicine, Mayo Clinic, Phoenix, AZ 85054, USA; hamad.abdullah@mayo.edu; 3 Division of Gastroenterology & Hepatology, Mayo Clinic, Phoenix, AZ 85054, USA; 4 Division of Internal Medicine, Mayo Clinic, Phoenix, AZ 85054, USA

**Keywords:** MASLD, MASH, aspirin, acetylsalicylic acid, NAFLD, NASH

## Abstract

**Background:** Given aspirin’s biologic plausibility for antifibrotic and antineoplastic effects, we conducted a systematic review and meta-analysis to examine the association between aspirin use and major liver-related outcomes in metabolic dysfunction-associated steatotic liver disease (MASLD). To our knowledge, this is the first systematic review and meta-analysis restricted exclusively to patients with biopsy- or registry-confirmed MASLD. **Methods:** A comprehensive search of Ovid MEDLINE, Ovid EMBASE, Scopus, and Web of Science was performed in October 2025. Studies enrolling adults with a confirmed MASLD diagnosis were included; those with viral hepatitis or alcohol-related liver disease were excluded. Outcomes assessed included hepatocellular carcinoma (HCC), fibrosis progression, cirrhosis, all-cause and liver-related mortality, gastrointestinal (GI) bleeding, hemorrhagic stroke, and liver disease progression. Hazard ratios (HRs) with 95% CIs were pooled using random-effects models. Heterogeneity was assessed using I^2^ statistics. **Results:** Seven studies met the eligibility criteria, with approximately 720,000 individuals included. Pooled analysis showed that aspirin use was associated with a significantly lower HCC risk (HR 0.59; 95% CI 0.43–0.81; I^2^ = 83%). No statistically significant association was found between aspirin use and cirrhosis incidence (HR 0.55; 95% CI 0.13–2.37; I^2^ = 83.4%) or GI bleeding (HR 1.11; 95% CI 0.74–1.66; I^2^ = 98.9%). Among the two studies that explored all-cause mortality, aspirin was associated with a modest but statistically significant reduction in all-cause mortality (HR 0.86; 95% CI 0.78–0.95; I^2^ = 0%). **Conclusions:** Aspirin use is associated with a reduced risk of HCC and all-cause mortality in MASLD without significantly increasing GI bleeding or hemorrhagic strokes. These associations may reflect aspirin’s anti-inflammatory properties in liver disease. Further RCTs are needed to verify the causal role of aspirin in MASLD management.

## 1. Introduction

In recent years, metabolic dysfunction-associated steatotic liver disease (MASLD) has emerged as a major global health concern [1]. Among adults in the United States (US), the prevalence of steatotic liver disease (SLD) is estimated to be around 37.9%, with MASLD representing approximately 86% of all SLD cases [2]. It is expected that, by 2050, MASLD prevalence will rise to 41.1%, affecting over 120 million adults in the US, doubling the incidence of hepatocellular carcinoma (HCC), tripling the prevalence of decompensated cirrhosis, and resulting in a quadruple increase in the need for liver transplantation compared to current estimates [2]. MASLD is also a growing cause of HCC. A meta-analysis of 64 observational studies estimated an overall HCC incidence of 1.25 per 1000 person-years in patients with MASLD, rising to approximately 20 per 1000 person-years in those with MASLD-related cirrhosis [3,4].

Management of MASLD requires a multidisciplinary approach given its wide spectrum of hepatic and extrahepatic manifestations [5,6]. Acetylsalicylic acid (aspirin) has shown promising effects in the management of MASLD with multiple studies demonstrating that aspirin use has been associated with lower hepatic steatosis, inflammation, fibrosis, cirrhosis, and HCC [7,8,9].

Although the HCC-preventive properties of aspirin have been explored in prior studies, most analyses included mixed liver disease populations with multiple etiologies such as viral hepatitis and alcohol-associated liver disease [10]. These designs limit the ability to draw conclusions specific to MASLD. Up to the present date, no meta-analysis has focused exclusively on the effect of aspirin in MASLD, strictly excluding other SLD etiologies and non-aspirin exposures. This meta-analysis aims to define the independent association between aspirin use and liver-related outcomes, particularly the risk of HCC, in patients with MASLD.

## 2. Methods

### 2.1. Search Strategy

The meta-analysis was conducted in accordance with the Preferred Reporting Items for Systematic Reviews and Meta-Analyses (PRISMA) 2020 framework [11]. The full PRISMA checklist is provided in Supplementary Table S1. The protocol was prospectively registered with PROSPERO (Registration ID: CRD420251150905). A comprehensive search was conducted across MEDLINE, EMBASE, Web of Science, and Scopus using aspirin-related terms combined with MASLD/MASH and liver cancer terms. Detailed search strategies for MEDLINE and EMBASE are provided in Supplementary Table S2; searches for the remaining databases followed equivalent keyword-based approaches adapted to each platform.

### 2.2. Primary and Secondary Outcomes

The primary outcome of the study was to explore the incidence of HCC among aspirin users in comparison to non-aspirin users. Secondary outcomes included incidence of cirrhosis, fibrosis progression, all-cause mortality, gastrointestinal (GI) bleeding, and hemorrhagic stroke.

### 2.3. Inclusion and Exclusion Criteria

Eligibility criteria were structured according to the PICO framework. Population (P): adults (≥18 years) with a confirmed diagnosis of MASLD established by histology, imaging, or validated diagnostic codes. Intervention (I): aspirin use, with exposure definitions varying across the included studies in dose, duration, and ascertainment (prescription records, self-report, or randomized assignment). Comparator (C): non-users. Outcomes (O): the primary outcome was incident HCC; studies reporting effect estimates with corresponding 95% confidence intervals (CI) for any secondary outcome were eligible for secondary analyses, regardless of whether HCC was assessed. Study design (S): observational cohort studies and randomized controlled trials (RCTs) published in English.

Studies were excluded if they did not report effect estimates [hazard ratio (HR), odds ratio (OR), or relative risk (RR) with 95% confidence intervals] for HCC or any of the secondary outcomes. Additional exclusion criteria included non-primary research (reviews, editorials, commentaries, conference abstracts, case reports), studies evaluating aspirin only in combination with other NSAIDs without a separable aspirin-only subgroup analysis, animal studies, and studies enrolling populations with viral hepatitis or alcohol-related liver disease unless MASLD-specific results were reported separately.

### 2.4. Study Selection and Data Extraction

All records identified through the search were screened independently by two investigators (F.J. and A.H.) in two stages: title/abstract screening followed by full-text review. Conflicts were resolved by a third reviewer (A.E.). No automation or machine-learning tools were used at any stage. Both screening and data extraction were performed independently and in duplicate, with reviewers blinded to one another’s decisions.

From each included study, the following information was extracted: publication year, country, study design, sample size, follow-up period, population characteristics, definition of aspirin exposure and dosing, comparator definition, outcomes assessed, effect estimates with 95% CI, and variables included in adjusted models.

When available, adjusted HRs were abstracted directly. When studies reported ORs or RRs for rare outcomes, these measures were treated as approximations of HRs, consistent with established epidemiologic principles when event rates are low.

### 2.5. Risk of Bias Assessment

The quality of observational cohort studies was assessed using the Newcastle–Ottawa Scale, evaluating study selection, comparability, and outcome ascertainment [12]. The randomized controlled trial was evaluated using the Cochrane Risk of Bias 2.0 tool [13].

Publication bias was examined only for the primary outcome (HCC incidence), as recommended for meta-analyses with at least three available studies using funnel plot visualization and Egger’s regression test.

### 2.6. Statistical Analysis

Statistical analyses were conducted in R version 4.3 using the meta package for meta-analytic pooling and forest plot generation and the metafor package for publication bias assessment. Effect sizes were pooled as HRs with 95% CIs. Random-effects models were applied for all endpoints using restricted maximum likelihood (REML) estimation to account for between-study variability.

Statistical heterogeneity was quantified using the χ^2^ test and the I^2^ statistic, with I^2^ > 50% or a *p*-value < 0.10 indicating substantial heterogeneity. When heterogeneity was present, a random-effects model using REML was applied. Given the expected clinical and methodological variability across real-world cohorts, the random-effects approach was selected a priori for all pooled analyses. Statistical significance was defined as a two-sided *p*-value < 0.05.

## 3. Results

A total of 794 records were identified, of which 30 duplicates were removed. Following title and abstract screening, 764 articles were assessed, of which 747 were excluded for not meeting the inclusion criteria. Seventeen full-text articles were reviewed in detail, and six were excluded because they were editorials, commentaries, or abstract-only publications. Of the remaining 11 full-text articles, four were excluded for the following reasons: evaluation of steatosis progression only (n = 1), mixed etiologies without exclusion of alcohol or viral hepatitis (n = 1), absence of confirmed MASLD diagnosis (n = 1), and cross-sectional design with no longitudinal outcomes (n = 1).

**Figure 1:**
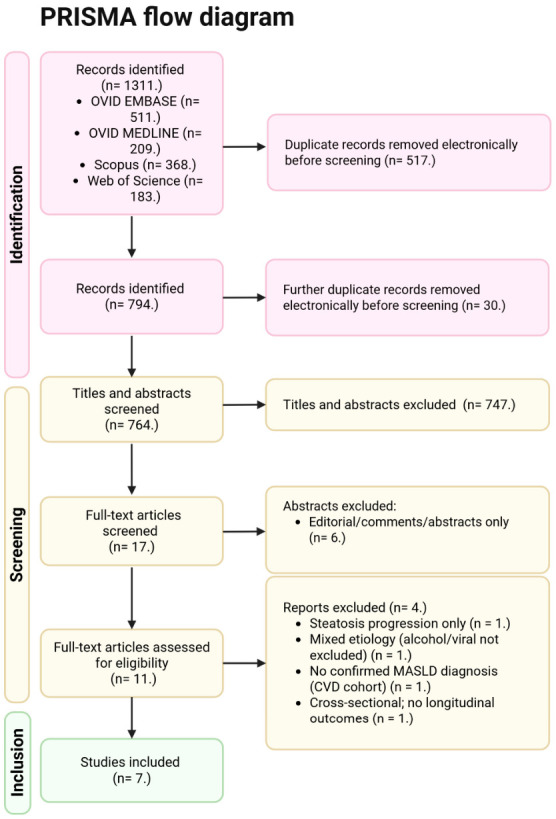
PRISMA flow diagram of study selection. Created in BioRender. Jamal, F. (2026). https://BioRender.com/gfj9tvq (accessed on 20 May 2026).

A total of seven studies met all the eligibility criteria and were included in the qualitative synthesis and meta-analysis (Figure 1) [7,14,15,16,17,18,19]. These studies collectively represented approximately 720,000 adults with confirmed MASLD and reported outcomes relevant to HCC, fibrosis progression, cirrhosis, mortality, and safety endpoints. Aspirin was most often defined as regular long-term use of low-dose aspirin (75–100 mg daily), with follow-up durations ranging from 6 months to over 11 years in large national cohorts. Table 1 summarizes the seven studies that met the inclusion criteria.

### 3.1. Primary Outcome: HCC

**Figure 2:**
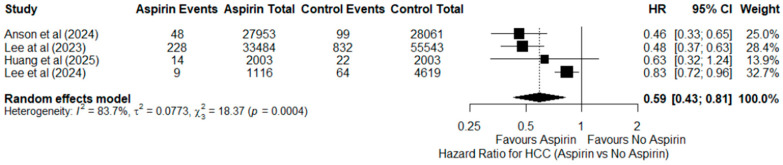
Forest plot of the association between aspirin use and hepatocellular carcinoma incidence in MASLD [14,17,18,19].

Four studies reported the association between aspirin use and incident HCC. A random-effects model demonstrated that aspirin use was associated with a significantly lower risk of HCC: HR 0.59 (95% CI 0.43–0.81; *p* = 0.001). Substantial heterogeneity was observed (I^2^ = 83.7%), which likely reflects differences in population characteristics, baseline fibrosis severity, exposure definitions, and analytic methods across large real-world cohorts. The direction of effect, however, was consistent across all studies (Figure 2). Publication bias assessment for HCC showed no evidence of small-study effects (Egger *p* = 0.63; Supplementary Figure S1). A summary of the pooled primary outcome, including heterogeneity statistics and Egger’s regression results, is provided in Table 2.

### 3.2. Secondary Outcomes

From the two studies that evaluated cirrhosis incidence, the pooled estimate showed no statistically significant association: HR 0.55 (95% CI 0.13–2.37) and I^2^ = 83.4% (Supplementary Figure S2). For all-cause mortality, aspirin use was associated with a modest but statistically significant reduction: HR 0.86 (95% CI 0.78–0.95) and I^2^ = 0% (Forest plot; Supplementary Figure S3). For GI bleeding, the pooled estimate from two studies demonstrated no significant increase in risk: HR 1.11 (95% CI 0.74–1.66) and I^2^ = 98.9% (Forest plot; Supplementary Figure S4). Only one study reported hemorrhagic stroke showing no increased risk among aspirin users: HR 1.07 (95% CI 0.95–1.20). Since only one study was available, meta-analysis was not performed. Two studies reported liver disease progression, but differing outcome definitions (paired liver biopsy vs. VCTE) precluded pooling. Both studies suggested potential antifibrotic benefit, including a RCT reporting a 2.8 kPa reduction in liver stiffness at 6 months with low-dose aspirin.

A summary of all secondary outcome meta-analytic estimates is provided in Supplementary Table S3.

## 4. Risk of Bias

Overall risk of bias was rated as low to moderate across the included studies. RCTs demonstrated low risk of bias across all domains, while observational studies were limited by residual confounding and heterogeneous outcome definitions (Table 3 and Table 4).

## 5. Discussion

This is the first meta-analysis studying the effect of aspirin on HCC, restricted to the MASLD population. Aspirin use was associated with a lower incidence of HCC among patients with MASLD (HR 0.59; 95% CI 0.43–0.81, *p*-value 0.001). Aspirin use was also associated with a statistically significant lower all-cause mortality in MASLD patients. Additionally, there was a pattern of lowering cirrhosis incidence among aspirin users, although it did not reach statistical significance. Regarding potential bleeding with aspirin, our results showed that there is a higher risk of GI bleeding and hemorrhagic stroke among the aspirin group, but the relation was not statistically significant.

Similar results were reflected in a meta-analysis by Tan et al. where aspirin use demonstrated a 49% reduction in HCC risk among patients with CLD [10]. Another nationwide Swedish cohort study reported a 43% reduction in HCC risk among patients with chronic viral hepatitis who used aspirin for five or more years [8]. The concordance of findings across distinct liver disease etiologies, including chronic viral hepatitis, MASLD, and alcoholic liver disease, suggests that aspirin may exhibit hepatoprotective effects [7,8,15,20]. By inhibition of the cyclooxygenase-2, an enzyme overexpressed in hepatic stellate cells and liver tumors; aspirin helps in reducing inflammation and tumor growth [21,22]. Additionally, recent data indicate that aspirin alters bile acid metabolism via SULT2A3 pathways, potentially lowering the risk of liver cancer development [23]. Aspirin also blocks platelet activation through glycoprotein Ibα pathways, which reduces platelet-mediated liver inflammation and fibrosis progression [24]. This has been supported by early clinical evidence, including a phase 2 randomized trial conducted by Simon et al. suggesting that aspirin use in patients with MASLD is associated with reduced disease progression to HCC [8].

The duration-dependent nature of aspirin’s association with HCC risk should be taken into consideration. Multiple studies report that lower HCC risk was associated with three to five years or more of continuous use. In the Swedish cohort, progressively lower HCC risk was observed with increasing duration of aspirin use (HR 0.90 for 1–3 years, HR 0.66 for 3–5 years, and HR 0.57 for ≥5 years) [8]. Collectively, these data suggest that exposure duration is likely an important effect modifier when evaluating aspirin’s potential hepatoprotective role.

In our analysis, aspirin use was not associated with a statistically significant increase in GI bleeding (HR 1.11; 95% CI 0.74–1.66) or hemorrhagic stroke (HR 1.07; 95% CI 0.95–1.20). The GI bleeding estimate was pooled from only two studies and showed very high heterogeneity (I^2^ = 98.9%), reflecting differences in bleeding definitions, follow-up, and concomitant antithrombotic, NSAID, and gastroprotective use, so it should be read with caution and does not exclude modest harm. In the general population, low-dose aspirin raises the relative risk of major GI bleeding by about 50% and of intracranial hemorrhage by 37% [25,26]. In cirrhosis, this risk is amplified by portal hypertension, thrombocytopenia, and impaired coagulation; in a US cohort of 18,070 cirrhosis patients, antiplatelet use was independently associated with more bleeding (aHR 1.31; 95% CI 1.00–1.72) and decompensation (aHR 1.44; 95% CI 1.06–1.95) [27,28]. Most patients in the included studies had non-cirrhotic or compensated MASLD, so these safety data apply poorly to advanced liver disease. Future trials should stratify bleeding outcomes by fibrosis stage, portal pressure, and platelet count to define the population in which aspirin can be used safely. Aspirin hepatotoxicity is dose-dependent and was historically described at high anti-inflammatory doses (typically ≥2 g/day), most often as reversible aminotransferase elevations [29]. The long-term hepatic safety of low-dose aspirin (75–100 mg/day) has not been examined directly. None of the studies included in this meta-analysis reported hepatotoxicity signals despite follow-up periods from six months to nearly eight years, although hepatic safety was not a primary or pre-specified endpoint in any of them [7,8,17]. Liver safety should be monitored as a prespecified endpoint in future trials of low-dose aspirin in MASLD. Despite these promising signals, current guidelines do not support aspirin use solely for HCC prevention. The American Association for the Study of Liver Diseases (AASLD) advises against use of aspirin, statins, and metformin solely to reduce HCC risk due to potential risks of toxicity and adverse events [30]. However, for MASLD patients already receiving aspirin for cardiovascular indications, a potential reduction in HCC risk may represent an added benefit.

## 6. Strengths and Limitations

This meta-analysis has several strengths. It is the first to systematically synthesize evidence on aspirin use and liver outcomes specifically in patients with MASLD, addressing a gap in the literature where prior reviews combined heterogeneous liver disease etiologies. The pooled analysis of over 720,000 patients across diverse populations from the US, Europe, and Asia enhances the generalizability of the findings.

Several limitations require consideration. Six of the seven included studies were observational, introducing the potential for confounding by indication and residual confounding. Substantial heterogeneity was observed across studies, reflecting the need for cautious interpretation of the pooled estimates. Furthermore, a limited number of studies were available when subgroup analysis of outcomes was attempted. Most studies relied on administrative diagnostic codes rather than histologic confirmation of MASLD, and aspirin exposure was variably defined across studies. The clinical indication for aspirin was not reported in any of the observational studies, hindering a subgroup analysis by indication (e.g., primary vs. secondary cardiovascular prevention). Finally, our search was restricted to English language publications, which may have excluded relevant studies published in other languages and introduces a degree of language bias.

Adequately powered randomized trials with follow-up durations of at least five years are needed to establish causality and to define optimal dosing, timing of initiation, and patient selection criteria.

## 7. Conclusions

In this systematic review and meta-analysis restricted to patients with MASLD, aspirin use was associated with a significantly lower risk of HCC and reduced mortality without a statistically significant increase in GI bleeding or hemorrhagic stroke. These findings suggest that aspirin may offer hepatoprotective benefits in MASLD, particularly for patients already receiving it for cardiovascular indications. However, prospective randomized trials with longer follow-up are needed to confirm causality and to define the role of aspirin as a potential disease-modifying strategy in MASLD.

## Figures and Tables

**Table 1:** Summary of studies evaluating aspirin use in MASLD.

Author (Year)	Country	Study Design	Population	Sample Size Aspirin vs. Control	Follow-Up	Exposure Definition and Dose	Comparator	Key Outcomes Reported	Adjustment Variables
Simon et al. (2024) [7]	USA	Phase 2 RCT	MASLD without cirrhosis, 18–70 yrs	40 vs. 40	6 mo	Low-dose aspirin 81 mg daily	Placebo	Change in liver stiffness (VCTE kPa)	Adjusted for baseline stiffness
Lee et al. (2023) [14]	Taiwan	Nationwide retrospective cohort	NAFLD without viral or alcohol-related liver disease	33,484 vs. 55,543	Median 7.9 yrs	≥90 days daily aspirin ≤100 mg	<90 days total exposure	HCC incidence; GI bleed; intracranial bleed	Age, sex, cirrhosis, ALT, DM, HTN, CAD, CVA, arrhythmia, PVD, metformin, statins
Simon et al. (2019) [15]	USA	Prospective cohort	Adults with NAFLD, no viral or alcoholic liver disease	151 vs. 210	Median 7.4 yrs	Daily aspirin use (dose not specified)	Non-regular/never use	Fibrosis progression (≥1 stage)	Age, sex, BMI, DM, HTN, HLD, smoking, statin, metformin, biopsy interval
Vell et al. (2023) [16]	UK and USA	Retrospective cohort	Confirmed MASLD	56,684 vs. 91,338	Mean 11.8 yrs	Regular aspirin use (daily/weekly/monthly)	Non-users	Fibrosis/cirrhosis; GI bleed	Age, sex, BMI, ethnicity, DM, HTN, dyslipidemia, alcohol, smoking, medications
Huang et al. (2025) [17]	Taiwan	Retrospective cohort	Adults ≥ 18 yrs with MASLD	2003 vs. 2003 controls	3 yrs	Daily aspirin 75–100 mg	Non-users	Liver-related death; all-cause mortality	Age, sex, cirrhosis, ALT, DM, HTN, CAD, stroke, meds, viral status
Lee et al. (2024) [18]	Taiwan	Nationwide retrospective cohort	MAFLD/MASH population	9238 subset (1116 aspirin; 4619 controls)	3 yrs	≥90 days daily aspirin	Non-users	Cirrhosis incidence; HCC incidence; mortality	Age, sex, DM, HTN, HLD, CAD, stroke, CKD, obesity, viral hepatitis, meds
Anson et al. (2024) [19]	Multinational	Retrospective cohort	NAFLD patients	27,953 vs. 28,061	5 yrs	Aspirin ≥ 1 yr post-MASLD diagnosis	No antiplatelet use	HCC	Age, sex, ethnicity, DM, obesity, neoplasm history, CVD

ALT: alanine aminotransferase, BMI: body mass index, CAD: coronary artery disease, CVD: cardiovascular disease, CVA: cerebrovascular accident, DM: diabetes mellitus, GI: gastrointestinal, HCC: hepatocellular carcinoma, HLD: hyperlipidemia, HTN: hypertension, MASLD: metabolic dysfunction-associated steatotic liver disease, PVD: peripheral vascular disease, RCT: randomized controlled trial, USA: United States of America, VCTE: vibration-controlled transient elastography.

**Table 2:** Meta-analysis of aspirin use and risk of hepatocellular carcinoma.

Outcome	Number of Studies	Pooled Effect	95% CI	*p*-Value	Model Used	Heterogeneity (I^2^ %)	Publication Bias
HCC Incidence	4	HR = 0.59	0.43–0.82	0.001	Random-effects (REML)	78	No evidence of publication bias (Egger *p* = 0.63)

CI: confidence interval, HR: hazard ratio, HCC: hepatocellular carcinoma, I^2^: heterogeneity index, REML: restricted maximum likelihood, *p*: *p*-value.

**Table 3:** Risk of bias assessment for included observational studies.

Study	Selection (★ Max 4)	Comparability (★ Max 2)	Outcome (★ Max 3)	Total ★/9	Overall Risk of Bias
Simon et al., 2019 [15]	★★★★	★★	★★★	9	Low risk
Huang et al., 2025 [17]	★★★	★★	★★	7	Low–moderate risk
Lee et al., 2023 [14]	★★★★	★★	★★	8	Low risk
Vell et al., 2023 [16]	★★★	★★	★★	7	Low–moderate risk
Lee et al., 2024 [18]	★★★★	★★	★★	8	Low risk
Anson et al., 2024 [19]	★★★	★★	★★	7	Low–moderate risk

★: resembles a point gained to the study, with more stars yielding a lower risk of bias.

**Table 4:** Risk of bias assessment for included randomized control trial.

Domain	Judgment	Justification
Bias arising from the randomization process	Low risk	The study used computer-generated randomization with concealed allocation. Baseline characteristics between aspirin and placebo groups were balanced.
Bias due to deviations from intended interventions	Low risk	Participants and investigators were blinded to treatment assignment. Adherence was monitored, and analyses were performed according to the intention-to-treat principle.
Bias due to missing outcome data	Low risk	Outcome data (liver stiffness measurements at 6 months) were available for >95% of randomized participants; missingness was minimal and unlikely to bias results.
Bias in measurement of the outcome	Low risk	Liver stiffness (kPa) was measured using standardized VCTE by trained personnel blinded to treatment group. Measurement methods were objective and validated.
Bias in selection of the reported result	Low risk	The study protocol prespecified liver stiffness change as the primary outcome. Reported analyses were consistent with the trial protocol. No evidence of selective reporting.
Overall risk of bias	Low risk	All domains were judged at low risk of bias, indicating high methodological quality.

kPa: kilopascal, VCTE: vibration-controlled transient elastography.

## Data Availability

Data used in this study were obtained from previously published studies cited within the manuscript and Supplementary Materials. No new datasets were generated. Extracted data are available from the corresponding author on request.

## Supplementary Materials

The following supporting information can be downloaded at https://www.mdpi.com/article/10.3390/biomedicines14061249/s1: Supplementary Table S1: PRISMA checklist. Supplementary Table S2: Detailed search strategy. Supplementary Figure S1: Funnel plot for HCC. Supplementary Figure S2: Cirrhosis incidence forest plot. Supplementary Figure S3: All-cause mortality forest plot. Supplementary Figure S4: GI bleeding forest plot. Supplementary Table S3: Summary of secondary outcomes. Reference [31] is cited in the Supplementary Materials.
