# Supplementary material for: Aspirin Use and Liver-Related Outcomes in Metabolic Dysfunction-Associated Steatotic Liver Disease: A Systematic Review and Meta-Analysis
Source: Biomedicines. 2026 May 30;14(6):1249. doi: 10.3390/biomedicines14061249 (PMC13296613; doi:10.3390/biomedicines14061249)
Supplement: Supplementary file 1 [file biomedicines-14-01249-s001.zip › biomedicines-4287304-supplementary.pdf]

Supplementary Table S1: The Preferred Reporting Items for Systematic Reviews and Meta-analysis (PRISMA) checklist.

Section/topic	#	Checklist item	Reported on page #
**TITLE**			
Title	1	Identify the report as a systematic review, meta-analysis, or both.	1
**ABSTRACT**			
Structured summary	2	Provide a structured summary including, as applicable: background; objectives; data sources; study eligibility criteria, participants, and interventions; study appraisal and synthesis methods; results; limitations; conclusions and implications of key findings; systematic review registration number.	1-2
**INTRODUCTION**			
Rationale	3	Describe the rationale for the review in the context of what is already known.	3-4
Objectives	4	Provide an explicit statement of questions being addressed with reference to participants, interventions, comparisons, outcomes, and study design (PICOS).	3-4
**METHODS**			
Protocol and registration	5	Indicate if a review protocol exists, if and where it can be accessed (e.g., Web address), and, if available, provide registration information including registration number.	4
Eligibility criteria	6	Specify study characteristics (e.g., PICOS, length of follow-up) and report characteristics (e.g., years considered, language, publication status) used as criteria for eligibility, giving rationale.	4-5
Information sources	7	Describe all information sources (e.g., databases with dates of coverage, contact with study authors to identify additional studies) in the search and date last searched.	4
Search	8	Present full electronic search strategy for at least one database, including any limits used, such that it could be repeated.	4
Study selection	9	State the process for selecting studies (i.e., screening, eligibility, included in systematic review, and, if applicable, included in the meta-analysis).	4-6
Data collection process	10	Describe method of data extraction from reports (e.g., piloted forms, independently, in duplicate) and any processes for obtaining and confirming data from investigators.	4-6
Data items	11	List and define all variables for which data were sought (e.g., PICOS, funding sources) and any assumptions and simplifications made.	4-6
Risk of bias in individual studies	12	Describe methods used for assessing risk of bias of individual studies (including specification of whether this was done at the study or outcome level), and how this information is to be used in any data synthesis.	6
Summary measures	13	State the principal summary measures (e.g., risk ratio, difference in means).	7
Synthesis of results	14	Describe the methods of handling data and combining results of studies, if done, including measures of consistency (e.g., I^2) for each meta-analysis.	7

Section/topic	#	Checklist item	Reported on page #
Risk of bias across studies	15	Specify any assessment of risk of bias that may affect the cumulative evidence (e.g., publication bias, selective reporting within studies).	6
Additional analyses	16	Describe methods of additional analyses (e.g., sensitivity or subgroup analyses, meta-regression), if done, indicating which were pre-specified.	7
**RESULTS**			
Study selection	17	Give numbers of studies screened, assessed for eligibility, and included in the review, with reasons for exclusions at each stage, ideally with a flow diagram.	7
Study characteristics	18	For each study, present characteristics for which data were extracted (e.g., study size, PICOS, follow-up period) and provide the citations.	7
Risk of bias within studies	19	Present data on risk of bias of each study and, if available, any outcome level assessment (see item 12).	10
Results of individual studies	20	For all outcomes considered (benefits or harms), present, for each study: (a) simple summary data for each intervention group (b) effect estimates and confidence intervals, ideally with a forest plot.	8-9
Synthesis of results	21	Present results of each meta-analysis done, including confidence intervals and measures of consistency.	8-9
Risk of bias across studies	22	Present results of any assessment of risk of bias across studies (see Item 15).	10
Additional analysis	23	Give results of additional analyses, if done (e.g., sensitivity or subgroup analyses, meta-regression [see Item 16]).	N/A
**DISCUSSION**			
Summary of evidence	24	Summarize the main findings including the strength of evidence for each main outcome; consider their relevance to key groups (e.g., healthcare providers, users, and policy makers).	10
Limitations	25	Discuss limitations at study and outcome level (e.g., risk of bias), and at review-level (e.g., incomplete retrieval of identified research, reporting bias).	13
Conclusions	26	Provide a general interpretation of the results in the context of other evidence, and implications for future research.	13
**FUNDING**			
Funding	27	Describe sources of funding for the systematic review and other support (e.g., supply of data); role of funders for the systematic review.	14

From: Moher D, Liberati A, Tetzlaff J, Altman DG, The PRISMA Group (2009). Preferred Reporting Items for Systematic Reviews and Meta-Analyses: The PRISMA Statement. PLoS Med 6(7): e1000097. doi:10.1371/journal.pmed1000097
For more information, visit: www.prisma-statement.org.

Supplementary Table S2. Detailed Search Strategy for Ovid MEDLINE and Ovid EMBASE

Electronic databases	Search	Search strategy	Results
**EMBASE**	#1	(masld or mash or nafld or nash).mp.	80535
	#2	("metabolic dysfunction–associated steatotic liver disease" or "metabolic dysfunction associated steatotic liver disease").mp.	6603
	#3	exp Nonalcoholic Fatty Liver/ or "non-alcoholic fatty liver disease*".mp.	89417
	#4	"non-alcoholic steatohepatitis".mp. or exp nonalcoholic steatohepatitis/	23816
	#5	1 or 2 or 3 or 4	110513
	#6	aspirin.mp. or exp acetylsalicylic acid/	287876
	#7	"antiplatelet agent*".mp.	13207
	#8	6 or 7	294440
	#9	5 or 8	698
	#10	limit 9 to (human and English language)	656
	#11	limit 10 to conference abstract	95
	#12	10 not 11	561
	#13	Limit 12 to animal studies	10
	#14	12 not 13	551
**MEDLINE**	#1	(masld or mash or nafld or nash).mp.	46234
	#2	("metabolic dysfunction–associated steatotic liver disease" or "metabolic dysfunction associated steatotic liver disease").mp.	4801
	#3	exp Non-alcoholic Fatty Liver Disease/ or "non-alcoholic fatty liver disease*".mp.	38857
	#4	("nonalcoholic fatty liver disease*" or "non-alcoholic fatty liver disease*").mp.	45775
	#5	("nonalcoholic steatohepatitis" or "non-alcoholic steatohepatitis").mp.	15694
	#6	1 or 2 or 3 or 4 or 5	60834
	#7	aspirin.mp. or exp Aspirin/	78273
	#8	"acetylsalicylic acid".mp.	1
	#9	"exp Platelet Aggregation Inhibitors/ or "antiplatelet agent*".mp.	145475
	#10	7 or 8 or 9	169016
	#11	6 and 10	321
	#12	Limit 11 to (English language and humans)	209

ASA: acetylsalicylic acid, HCC: hepatocellular carcinoma, MASLD: metabolic dysfunction–associated steatotic liver disease, MASH: metabolic dysfunction–associated steatohepatitis, NAFLD: non-alcoholic fatty liver disease, NASH: non-alcoholic steatohepatitis,

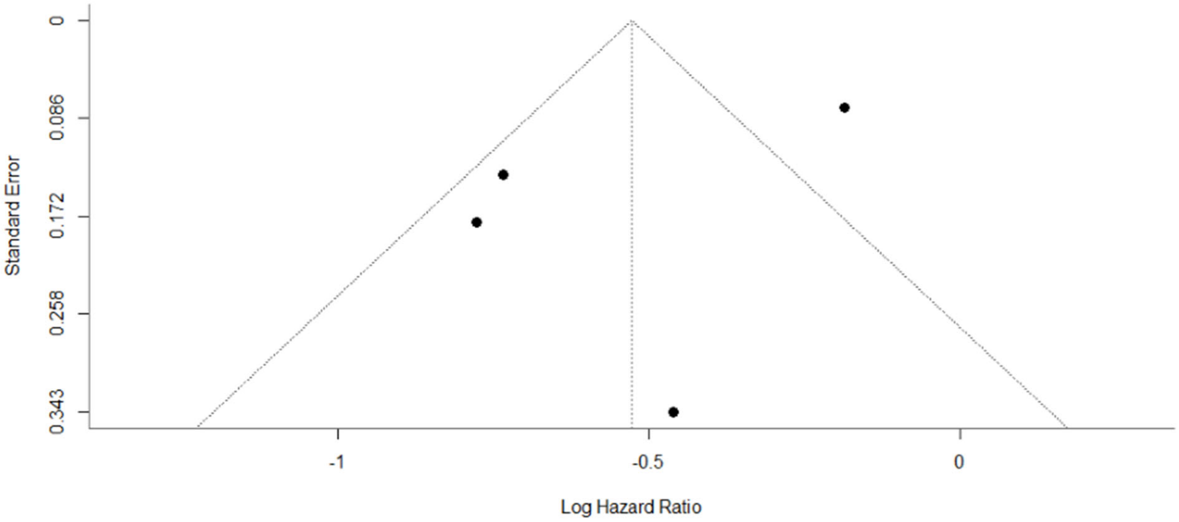

Supplementary Figure S1. Funnel plot for studies reporting hazard ratios for hepatocellular carcinoma (HCC). Circles represent individual study effect sizes plotted against their standard error. The vertical line indicates the pooled random-effects estimate.

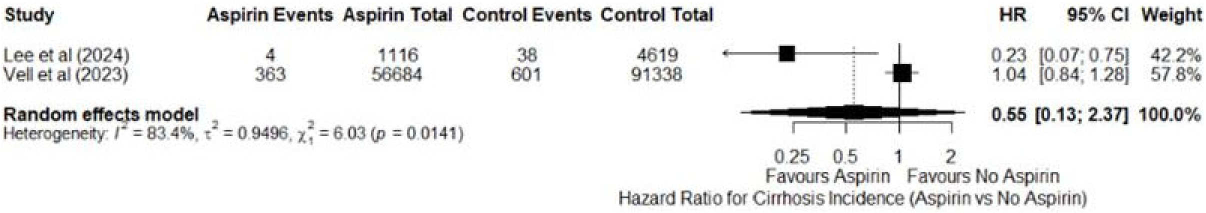

Supplementary Figure S2. Forest plot showing the pooled hazard ratio for incident cirrhosis among aspirin users versus non-users in MASLD.

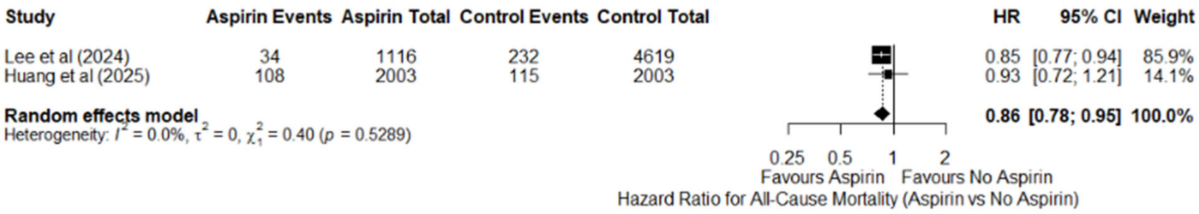

Supplementary Figure S3. Forest plot showing the pooled hazard ratio for incident all-cause mortality among aspirin users versus non-users in MASLD.

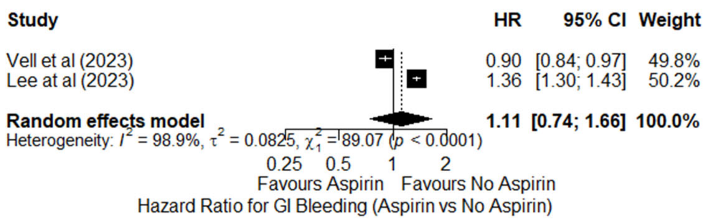

Supplementary Figure S4. Forest plot showing the pooled hazard ratio for incident gastrointestinal bleeding among aspirin users versus non-users in MASLD.

Supplementary Table S3. Summary of Secondary Outcomes Assessed in the Meta-analysis

Outcome	Number of Studies	Pooled Effect	95% CI	p-value	Model Used	Heterogeneity (I^2, %)	Publication Bias
Cirrhosis incidence	2	HR = 0.55	0.13–2.37	0.19	Random-effects (REML)	83.4	Not assessable (k=2)
All-cause mortality	2	HR = 0.86	0.78–0.95	0.004	Random-effects (REML)	0	Not assessable (k=2)
GI bleeding	2	HR = 1.11	0.74–1.66	0.63	Random-effects (REML)	98.9	Not assessable (k=2)
Hemorrhagic stroke	1	HR = 1.07	0.95–1.20	0.23	Not pooled	—	Not applicable
Liver disease progression	2	— (not pooled)	—	—	Not pooled	—	Not applicable

CI: confidence interval, HR: hazard ratio, HCC: hepatocellular carcinoma, I^2: heterogeneity index, REML: restricted maximum likelihood, p: p-value
